# Suicidal Ideations in Major Depressed Subjects: Role of the Temporal Dynamics of Anhedonia

**DOI:** 10.3390/brainsci13071065

**Published:** 2023-07-13

**Authors:** Gil Darquennes, Benjamin Wacquier, Gwenolé Loas, Matthieu Hein

**Affiliations:** Service de Psychiatrie et Laboratoire du Sommeil, Hôpital Universitaire de Bruxelles, Université libre de Bruxelles (ULB), Route de Lennik, 808-1070 Anderlecht, Belgium; gil.darquennes@ulb.ac.be (G.D.); benjamin.wacquier@hubruxelles.be (B.W.); gwenole.loas@hubruxelles.be (G.L.)

**Keywords:** anhedonia, suicidal ideations, major depression, epidemiology

## Abstract

Given the limited data available in the literature, the aim of this study was to investigate the potential role played by the temporal dynamics of anhedonia (lifelong anhedonia and recent changes in anhedonia) in the occurrence of suicidal ideations in major depressed subjects. The clinical data of 285 major depressed subjects recruited from the database of the Erasme Hospital Sleep Laboratory were analyzed. A score on item nine of the Beck Depression Inventory (BDI-II) ≥1 and/or an identification during the systematic psychiatric assessment were used to determine the presence of suicidal ideations. The association between anhedonia complaints (lifelong anhedonia and recent change in anhedonia) and suicidal ideations in major depressed subjects was assessed by logistic regression analyzes. The prevalence of suicidal ideations was 39.3% in our sample of major depressed subjects. After adjusting for the main confounding factors, multivariate logistic regression analysis demonstrated that unlike lifelong anhedonia, only recent changes in anhedonia were a risk factor for suicidal ideations in major depressed subjects. Given this potential involvement of the recent change in anhedonia in the occurrence of suicidal ideations in major depressed subjects, it seems essential to better identify and adequately manage this specific form of anhedonia in order to open new perspectives for the prevention of suicide in this particular sub-population.

## 1. Introduction

Given recent data from the World Health Organization, suicide remains an important public health problem despite the various prevention strategies implemented in recent years [1]. Indeed, the annual number of deaths by suicide is estimated at 700,000 worldwide, and suicide remains one of the leading causes of death among 15–29 year-olds [1]. In the general population, major depressive disorder is one of the main risk factors for suicide since this psychiatric disorder is very frequently found during psychological autopsies in subjects who died by suicide [2,3]. However, in major depressed subjects, the occurrence of suicidal behavior (suicide attempt and suicide) is generally preceded by the development of suicidal ideations, which is a marker of the severity of major depressive episodes [4,5]. Nevertheless, despite a high prevalence (37.7%), suicidal ideations are frequently underdiagnosed in major depressed subjects, although they are associated with suicidal acts in 15% of cases [6,7,8]. Thus, since suicidal ideations seem to be the first step of the suicidal plan in major depressed subjects [9], it is essential to identify the potential factors involved in the occurrence of these suicidal ideations in order to better prevent excess mortality by suicide in this particular population.

In the literature, the available studies seem to indicate that some specific depressive symptoms play a central role in the development of suicidal ideations [10]. Among these, depressive symptoms are associated with a more frequent occurrence of suicidal ideations, and anhedonia is characterized by an inability to feel positive emotions during life situations that were previously considered pleasant [11]. However, despite the fact that anhedonia is a cardinal symptom of major depressive disorder [12], few studies have investigated the role played by this depressive symptom in the occurrence of suicidal ideations in major depressed subjects [13,14,15]. Moreover, unlike other specific subpopulations [16,17,18], the potential impact of the temporal dynamics of anhedonia (lifelong anhedonia and recent changes in anhedonia) on the risk of suicidal ideations has been few studied in major depressed subjects [19], which could limit the interpretation of most of the studies available in the literature. Thus, given these limited data, it seems necessary to carry out additional investigations to better understand the potential role played by the temporal dynamics of anhedonia (lifelong anhedonia and recent changes in anhedonia) in the occurrence of suicidal ideations in major depressed subjects.

The aim of this study was to investigate the risk of suicidal ideations associated with lifelong anhedonia and recent changes in anhedonia in a large sample of major depressed subjects. Our hypothesis was that, similar to other specific subpopulations, only recent changes in anhedonia are associated with a higher risk of suicidal ideations in major depressed subjects. The objective of this approach was to provide healthcare professionals with reliable data regarding the potential role played by the temporal dynamics of anhedonia (lifelong anhedonia and recent changes in anhedonia) in the occurrence of suicidal ideations in major depressed subjects to enable better prevention of mortality by suicide in this particular subpopulation.

## 2. Materials and Methods

### 2.1. Population

**Figure 1:**
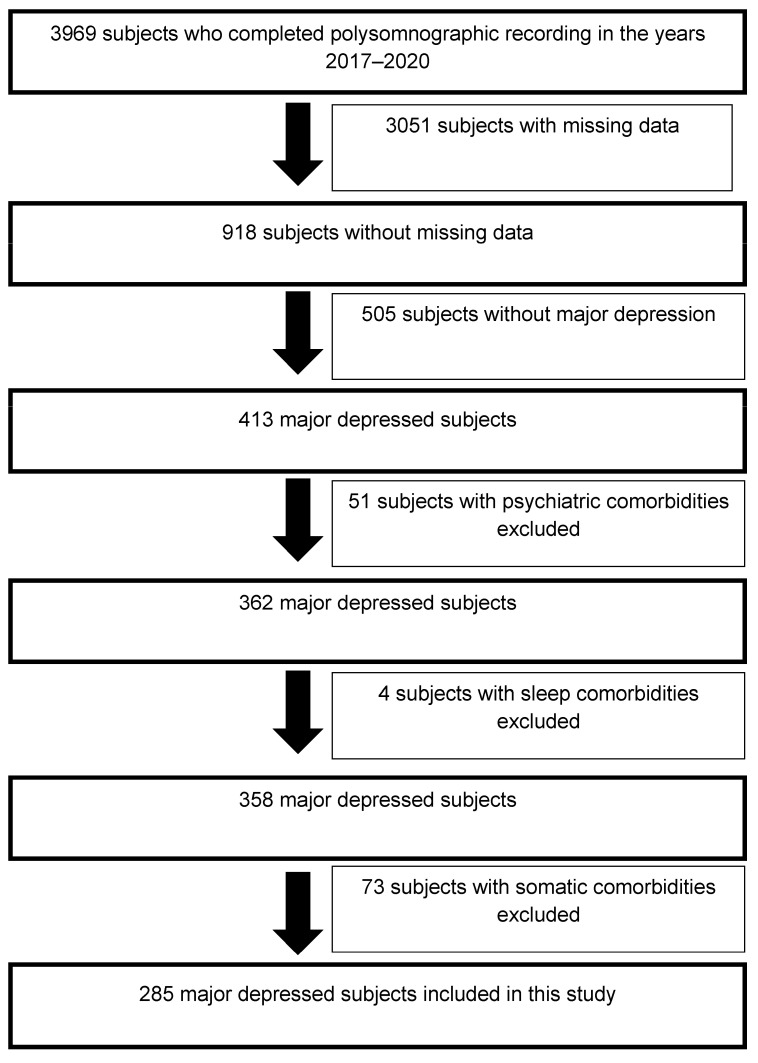
Selection diagram of major depressed subjects included in this study.

The data of 285 major depressed subjects who carried out a polysomnographic recording between 1 January 2017 and 31 December 2020 were collected from the database of the Erasme Hospital Sleep Laboratory (Figure 1). Table 1 presents the inclusion and exclusion criteria applied for the recruitment of these major depressed subjects. In addition, in this study, we only recruited major depressed subjects since the objective was to focus on this particular subpopulation where anhedonia complaints seem to play a central role in the occurrence of suicidal ideations [13,14,15,19]. Finally, Annex S1 (Supplementary Data) describes the outpatient recruitment procedure for the major depressed subjects included in this study.

### 2.2. Medical, Psychiatric, and Sleep Assessments of Major Depressed Subjects

The description of these different assessments and their objectives is available in Table 2. Regarding the assessment of anhedonia complaints (lifelong anhedonia and recent changes in anhedonia) and type D personality, three specific scales were used:

- Recent changes in anhedonia were investigated by the Anhedonia subscale of Beck Depression Inventory (items 4, 12, and 21 of the Beck Depression Inventory [BDI-II]) [20]. However, given the absence of validated cut-offs for this Anhedonia subscale of Beck Depression Inventory, recent changes in anhedonia were considered present when the score of this subscale was >3, which seemed to be most consistent with the psychometric properties of this subscale demonstrated in the article by Joiner et al. (2003) [20].
- Lifelong anhedonia was investigated by the Temporal Pleasure Experience Scale (TEPS) (Annex S2—Supplementary Data) [21]. However, given the absence of validated cut-offs for TEPS, lifelong anhedonia was considered present when the score on this scale was <76, which corresponded to the median of the TEPS distribution in our study.
- The presence of type D personality (stable personality structure characterized by negative affectivity and social inhibition) was assessed with the Type-D Personality Scale (DS14). This scale consists of 14 items that may be scored from 0 to 4. It is subdivided into 2 subscales of 7 items: a negative affectivity scale and a social inhibition scale. A score ≥10 on each subscale indicates the presence of type D personality [22].

Based on the psychiatric assessment and the self-questionnaires, the potential presence of suicidal ideations was determined in all major depressed subjects. Indeed, suicidal ideations were considered present if the score on item 9 of the Beck Depression Inventory (BDI-II) was ≥1 and/or if they were highlighted during the systematic psychiatric assessment [16,23,24].

**Table 2:** Description of medical, psychiatric, and sleep assessments.

	Different Assessments	Objectives
Medical assessment	Review of the medical record Complete somatic check-up (including blood test, electrocardiogram, and daytime electroencephalogram)	Systematic diagnosis of potential somatic comorbidities
Psychiatric assessment	Standardized semi-structured psychiatric interview based on DSM 5 diagnostic criteria [9] specific to Erasme Hospital Sleep Laboratory conducted by a junior psychiatrist and supervised by a senior psychiatrist to guarantee the best possible reliability of psychiatric diagnoses	Systematic diagnosis of potential psychiatric comorbidities
Self-questionnaires	Beck Depression Inventory (BDI-II) Spielberger Anxiety Inventory (state–trait) Insomnia Severity Index Epworth Sleepiness Scale	Assessment of subjective complaints of depression, anxiety, insomnia, and daytime sleepiness (Annex S2—Supplementary Data)
Sleep assessment	Semi-structured sleep interview Polysomnographic recording	Systematic diagnosis of potential comorbid sleep disorders according to the diagnostic criteria of the American Academy of Sleep Medicine [25] (Annex S3—Supplementary Data)

### 2.3. Statistical Analyzes

Stata software (version 14) was used to perform the statistical analyzes. Histograms, boxplots, and quantile-quantile plots were performed to control for the distribution of the data, whereas Levene’s tests were performed to control for the equality of variances.

Based on the criteria used in this study to identify the presence of suicidal ideations in major depressed subjects, a control group without suicidal ideations and a patient group with suicidal ideations were defined to allow for the different analyzes.

Given the asymmetrical distribution of most continuous data, their medians (P25–P75) were used for descriptive analyzes, and non-parametric tests (Wilcoxon test) were used for comparative analyzes. Concerning categorical data, percentages were used for descriptive analyzes and Chi² tests were used for comparative analyzes.

Univariate logistic regression models were used to study the risk of suicidal ideations associated with anhedonia complaints and potential confounding factors (Annex S4—Supplementary Data). Methodologically, even in the case of pre-existing lifelong anhedonia, all major depressed subjects with an Anhedonia subscale of Beck Depression Inventory score of >3 were included in the “recent change of anhedonia” group, given the occurrence of a recent change in their anhedonia complaints compared to their pre-morbid state [16]. In multivariate logistic regression models, the risk of suicidal ideations associated with anhedonia complaints was only adjusted for significant confounders in univariate analyzes. These different confounding factors were introduced hierarchically into the different multivariate models.

The Hosmer and Lemeshow test was performed to control for the adequacy of the final model, whereas the Link test was performed to control for the specificity of the final model.

A *p*-value of < 0.05 was used to identify significant results.

## 3. Results

### 3.1. Univariate Analyzes

The prevalence of suicidal ideations was 39.3% (*n* = 112) in our sample of major depressed subjects. Age, use of benzodiazepine receptor agonists, use of antidepressant therapy, presence of anxiety symptoms, depression severity, presence of type D personality, and anhedonia complaints were significantly associated with a higher risk of suicidal ideations in major depressed subjects. Additionally, compared to those without suicidal ideation, major depressed subjects with suicidal ideations had higher scores on the Beck Depression Inventory (BDI-II), the Anhedonia subscale of Beck Depression Inventory, the Beck Depression Inventory (BDI-II) reduced to 17 items (without items 4, 9, 12, and 21), the Spielberger Anxiety Inventory–Trait, the Spielberger Anxiety Inventory–State, the Type-D Personality Scale (DS-14), the Type-D Personality Subscale–Negative Affectivity, and Type-D Personality Subscale–Social Inhibition. The two groups of major depressed subjects did not differ significantly for the other demographic parameters (Table 3).

### 3.2. Multivariate Regression Analysis

After adjusting for the main significant confounding factors during the univariate analysis, multivariate logistic regression analyzes demonstrate that unlike lifelong anhedonia, only recent changes in anhedonia were significantly associated with a higher risk of suicidal ideations in major depressed subjects (Table 4).

### 3.3. Polysomnographic Data

Compared to those without suicidal ideations, major depressed subjects with suicidal ideations had prolonged REM latency. The two groups of major depressed subjects did not differ significantly for the other polysomnographic parameters (Table 5).

## 4. Discussion

In this study, we have shown that suicidal ideations were present in 39.3% of the major depressed subjects in our sample. However, this prevalence seems to be lower than that of some studies investigating the specific relationship between anhedonia and suicidal ideations in major depressed subjects. Indeed, in the study by Xie et al. (2014) [14], 67.5% of major depressed subjects had suicidal ideations, which could be explained by the fact that unlike our study, where major depressed subjects were recruited from an outpatient consultation, all major depressed subjects included in the study by Xie et al. (2014) were recruited from psychiatric units [14]. However, in the literature, it has been shown that hospitalized major depressed subjects have a higher prevalence of suicidal ideations than ambulatory major depressed subjects [6,26,27]. On the other hand, the prevalence of suicidal ideation highlighted in our sample of major depressed subjects seems to be consistent with that of other studies investigating the association between anhedonia complaints and the occurrence of suicidal ideations in major depressed subjects. Indeed, in the study by Ducasse et al. (2020), the prevalence of suicidal ideations was 40.6% in subjects with mood disorders [15]. However, similar to our study, all subjects with mood disorders included in the study by Ducasse et al. (2020) were recruited from an outpatient consultation [15], which could explain this concordance in the prevalence of suicidal ideations in our respective samples of patients. Thus, similar to the available literature [28], we have demonstrated that suicidal ideations are a significant problem in major depressed subjects recruited from an outpatient consultation, which seems to justify a better identification of their specific risk factors in this particular subpopulation.

Consistent with the literature [29,30], we demonstrated that anhedonia complaints were frequent (67.7%) in major depressed subjects. Indeed, lifelong anhedonia and recent changes in anhedonia were present in 26.0% and 41.7% of the major depressed subjects in our sample, respectively. Additionally, similar to other specific subpopulations [16,17,31,32], we demonstrated that unlike lifelong anhedonia, only recent changes in anhedonia were associated with a higher risk of suicidal ideations in major depressed subjects. Pathophysiologically, several elements could help to better understand this high prevalence of recent changes in anhedonia and their implication in the development of suicidal ideations in major depressed subjects. First, in major depressed subjects, there is evidence for reduced activity of the nucleus accumbens and the anterior cingulate cortex, which plays a central role in the normal functioning of the reward circuit [33,34]. However, these alterations in the normal functioning of the reward circuit induced by major depression may be associated with an aberrant treatment of the reward phenomenon favoring the occurrence of a reduced reactivity to experiences generating pleasure [33,34], which could explain the high prevalence of recent changes in anhedonia demonstrated in our sample of major depressed subjects. Second, the inability to respond to positive internal and external stimuli associated with recent changes in anhedonia may promote the emergence of major psychological pain [14,35]. However, in order to escape this major psychological pain, subjects with recent changes in anhedonia seem to tend to develop avoidance strategies characterized by the occurrence of suicidal ideations [14,35], which could explain the increased risk of suicidal ideations associated with recent changes in anhedonia highlighted in our sample of major depressed subjects. Third, there are differences in the temporal dynamics of lifelong anhedonia and recent changes in anhedonia. Indeed, the chronic inability to respond to positive internal and external stimuli associated with lifelong anhedonia is relatively stable over time, whereas the diminished responsiveness to pleasure-generating experiences associated with recent changes in anhedonia corresponds to a transient disruption of normal pre-morbid functioning by somatic and/or psychiatric pathologies [36]. However, these distinct temporal dynamics, according to the type of anhedonia complaints, seem to be associated with a more frequent development of psychological pain avoidance strategies characterized by the occurrence of suicidal ideations in subjects with recent changes in anhedonia than in those with lifelong anhedonia [37], which could help to better understand the lack of association between lifelong anhedonia and suicidal ideations in our study. Thus, based on these different elements, a better identification of the recent changes in anhedonia seems to be necessary in major depressed subjects in order to allow the establishment of more targeted, preventive, and therapeutic strategies in this subpopulation at high risk of suicide.

Although conventional treatments for major depression may improve complaints of suicidal ideations [38], highlighting this higher risk of suicidal ideations associated with recent changes in anhedonia in our sample of major depressed subjects could open new therapeutic perspectives for better management of suicidal ideations in this particular subpopulation. Indeed, given the potential central role played by this specific form of anhedonia in the occurrence of suicidal ideations in major depressed subjects, the establishment of therapeutic strategies targeting anhedonia that are complementary to conventional treatments for major depression could be an interesting option to reduce complaints of suicidal ideations in this particular subpopulation [39]. However, among the therapeutic strategies targeting anhedonia currently available, some pharmacological treatments (intravenous ketamine and intranasal esketamine) and some specific psychotherapeutic interventions (Positive affect treatment) seem to show promising results for both the improvement of depressive symptoms and suicidal ideations in anhedonic major depressed subjects [40,41,42]. Finally, independently of this potential positive impact of therapeutic strategies targeting anhedonia on suicidal ideations in major depressed subjects, it remains essential to respect the treatment recommendations for major depression in order to avoid the maintenance of residual depressive symptoms that may promote the persistence of suicidal ideations in this particular subpopulation [43,44,45].

### Limitations

Since the data used were extracted retrospectively without direct verification with the recruited subjects, additional prospective studies are needed to confirm the results obtained in this study. Furthermore, we only focused on anhedonia, which means that our results cannot be generalized to other affective symptoms. Moreover, since we only included major depressed subjects, our results are not generalizable to subjects with other psychiatric disorders, which may potentially limit their interpretation. On the other hand, given the inability to ensure sufficient surveillance and the presence of multiple cables in the rooms of the Erasme Hospital Sleep Laboratory, subjects with active suicidal ideations at high risk of suicidal behaviors are not admitted to this unit to avoid any risk of suicidal act but are referred to the Psychiatry Department to benefit from adequate management. However, this exclusion of subjects with active suicidal ideations at high risk of suicidal behaviors could limit the generalization of our findings to all major depressed subjects with suicidal ideations. Finally, our database only contains major depressed subjects who agreed to perform a polysomnographic recording, which may limit the generalizability of our results.

## 5. Conclusions

In this study, we confirmed that the prevalence of suicidal ideations is high (39.3%) in major depressed subjects. Furthermore, we demonstrated that unlike lifelong anhedonia, only recent changes in anhedonia were associated with a higher risk of suicidal ideations in major depressed subjects, which seems to justify better identification and adequate management of this specific form of anhedonia in order to open new perspectives for the prevention of suicide in this particular subpopulation.

## Figures and Tables

**Table 1:** Inclusion and exclusion criteria.

Inclusion Criteria	Exclusion Criteria
Patients (≥18 years) with moderate to severe major depressive episodes meeting the diagnostic criteria of DSM-5 [9]	Presence of psychiatric disorders other than major depression, active suicidal ideations with a high risk of suicidal behaviors, or abuse of substances
Absence of current severe uncontrolled somatic pathologies affecting the cardiopulmonary, digestive, renal, neurologic, endocrine, or immune systems	Presence of structural or functional brain lesions secondary to cranial trauma or neurological pathologies
Absence of craniofacial anomalies or thoracic malformations	Presence of central hypersomnia, current parasomnia, or sleep apnea syndrome with a predominantly central component
	Pregnancy

**Table 3:** Univariate analyzes (*n* = 285).

Variables	Categories	%	Major Depression without Suicidal Ideation	Major Depression with Suicidal Ideation	*p*-Value Chi^2^	OR (CI 95%)	*p*-Value
Gender	Female (*n* = 162) Male (*n* = 123)	56.8% 43.2%	56.1% 43.9%	58.0% 42.0%	0.743	1 0.92 (0.57 to 1.49)	0.743
BMI (kg/m^2^)	<25 (*n* = 84) ≥25 and <30 (*n* = 98) ≥30 (*n* = 103)	29.5% 34.4% 36.1%	31.2% 32.4% 36.4%	26.8% 37.5% 35.7%	0.612	1 1.35 (0.74 to 2.46) 1.14 (0.63 to 2.08)	0.613
Age (years)	30–45 (*n* = 112) <30 (*n* = 58) >45 (*n* = 115)	39.3% 20.4% 40.3%	45.7% 19.1% 35.2%	29.5% 22.3% 48.2%	0.021	1 1.81 (0.94 to 3.51) 2.12 (1.23 to 3.66)	0.022
Benzodiazepine receptor agonists	No (*n* = 227) Yes (*n* = 58)	79.6% 20.4%	85.5% 15.5%	70.5% 29.5%	0.002	1 2.47 (1.37 to 4.45)	0.003
Antidepressant therapy	No (*n* = 196) Yes (*n* = 89)	68.8% 31.2%	76.3% 23.7%	57.1% 42.9%	0.001	1 2.41 (1.45 to 4.03)	0.001
Other psychotropic treatments	No (*n* = 237) Yes (*n* = 48)	83.2% 16.8%	85.5% 15.5%	79.5% 20.5%	0.180	1 1.53 (0.82 to 2.86)	0.182
Smoking	No (*n* = 221) Yes (*n* = 64)	77.5% 22.5%	79.2% 20.8%	75.0% 25.0%	0.408	1 1.27 (0.72 to 2.23)	0.408
Alcohol	No (*n* = 151) Yes (*n* = 134)	53.0% 47.0%	53.2% 46.8%	52.7% 47.3%	0.934	1 1.02 (0.63 to 1.64)	0.934
Somatic treatments	No (*n* = 146) Yes (*n* = 139)	51.2% 48.8%	52.0% 48.0%	50.0% 50.0%	0.739	1 1.08 (0.67 to 1.74)	0.739
OSAS	No (*n* = 164) Mild (*n* = 60) Moderate to severe (*n* = 61)	57.5% 21.1% 21.4%	57.2% 20.8% 22.0%	58.1% 21.4% 20.5%	0.958	1 1.02 (0.56 to 1.86) 0.92 (0.50 to 1.69)	0.958
Sleep duration (hours)	≥6 (*n* = 187) <6 (*n* = 98)	65.6% 34.4%	63.6% 36.4%	68.8% 31.2%	0.370	1 0.79 (0.48 to 1.32)	0.370
Sleep movement disorders	No (*n* = 223) Yes (*n* = 62)	78.2% 21.8%	82.1% 17.9%	72.3% 27.7%	0.051	1 1.75 (0.99 to 3.09)	0.053
Excessive daytime sleepiness	No (*n* = 127) Yes (*n* = 158)	44.6% 55.4%	42.2% 57.8%	48.2% 51.8%	0.318	1 0.78 (0.49 to 1.26)	0.318
Insomnia Severity Index	<15 (*n* = 81) ≥15 (*n* = 204)	28.4% 71.6%	29.5% 70.5%	26.8% 73.2%	0.622	1 1.14 (0.67 to 1.94)	0.622
Anxiety symptoms	No (*n* = 108) Trait anxiety alone (*n* = 38) State anxiety alone (*n* = 45) Trait + state anxiety (*n* = 94)	37.9% 13.3% 15.8% 33.0%	46.8% 11.0% 17.9% 24.3%	24.1% 17.0% 12.5% 46.4%	<0.001	1 3.00 (1.39 to 6.48) 1.35 (0.63 to 2.92) 3.71 (2.05 to 6.74)	<0.001
BDI (17 items)	<15 (*n* = 76) ≥15 (*n* = 209)	26.7% 73.3%	31.8% 68.2%	18.8% 81.2%	0.015	1 2.02 (1.14 to 3.58)	0.016
Type D personality	No (*n* = 120) Yes (*n* = 165)	42.1% 57.9%	50.9% 49.1%	28.6% 71.4%	<0.001	1 2.59 (1.56 to 4.30)	<0.001
Anhedonia	No (*n* = 92) Lifelong (*n* = 74) Recent change (*n* = 119)	32.3% 26.0% 41.7%	40.5% 26.0% 33.5%	19.6% 25.9% 54.5%	<0.001	1 2.05 (1.05 to 4.00) 3.35 (1.84 to 6.09)	<0.001
Suicidal ideation	No (*n* = 173) Yes (*n* = 112)	60.7% 39.3%					
	**Median** **(P25–P75)**				**Wilcoxon test**		
BMI (kg/m^2^	27.8 (24.2–32.0)		28.1 (23.9–32.9)	27.7 (24.7–31.2)	0.683		
Age (years)	42 (32–52)		41 (33–51)	44 (31–54)	0.374		
ESS	11 (7–14)		11 (8–14)	11 (7–14)	0.462		
BDI	20 (17–27)		18 (16–22)	26 (19–33)	<0.001		
BDI–anhedonia	3 (2–4)		3 (1–4)	4 (2–5)	<0.001		
BDI (17 items)	17 (14–23)		16 (14–20)	22 (15–27)	<0.001		
ISI	17 (14–21)		17 (13–21)	18 (14–20)	0.733		
Spielberger Anxiety Inventory–Trait	52 (46–58)		50 (45–56)	56 (49–63)	<0.001		
Spielberger Anxiety Inventory–State	45 (36–54)		42 (35–49)	51 (40–59)	<0.001		
TEPS	76 (68–85)		78 (68–85)	74 (67–82)	0.072		
TEPS–Anticipatory	40 (34–46)		41 (35–46)	38 (33–44)	0.065		
TEPS–Consummatory	37 (30–41)		37 (31–42)	36 (30–40)	0.148		
DS-14	28 (20–36)		25 (18–32)	34 (26–41)	<0.001		
DS–Negative affectivity	16 (11–19)		13 (9–17)	18 (15–22)	<0.001		
DS–Social inhibition	13 (7–19)		12 (6–17)	16 (10–21)	0.001		

BMI = body mass index, OSAS = obstructive sleep apnea syndrome, BDI = Beck depression inventory, TEPS = temporal experience of pleasure scale, ESS = Epworth sleepiness scale, ISI = insomnia severity index, DS = type-D personality scale.

**Table 4:** Multivariate analysis (*n* = 285).

Variables	Model 1 OR Adjusted (CI 95%)	*p*-Value	Model 2 OR Adjusted (CI 95%)	*p*-Value	Model 3 OR Adjusted (CI 95%)	*p*-Value	Model 4 OR Adjusted (CI 95%)	*p*-Value
Anhedonia No lifelong Recent change	1 1.98 (1.00 to 3.89) 3.46 (1.88 to 6.38)	<0.001	1 2.11 (1.05 to 4.23) 3.15 (1.68 to 5.91)	0.002	1 1.75 (0.86 to 3.57) 2.58 (1.35 to 4.95)	0.017	1 1.85 (0.89 to 3.84) 2.35 (1.21 to 4.57)	0.041

Model 1 = model adjusted for age; Model 2 = model adjusted for age, benzodiazepine receptor agonists, and antidepressant therapy; Model 3 = model adjusted for age, benzodiazepine receptor agonists, antidepressant therapy, and type D personality; Model 4 = model adjusted for age, benzodiazepine receptor agonists, antidepressant therapy, type D personality, anxiety symptoms, and depression severity.

**Table 5:** Polysomnographic data (*n* = 285).

	Whole Sample	Major Depression without Suicidal Ideation	Major Depression with Suicidal Ideation	*p*-Value
Sleep latency (min)	57.0 (30.5–102.0)	58.0 (33.5–106.0)	55.0 (28.0–93.5)	0.150
Sleep efficiency (%)	75.3 (65.8–82.9)	74.2 (65.9–83.0)	75.9 (65.2–82.7)	0.432
Sleep period time (min)	438.5 (401.5–473.0)	438.5 (391.5–470.5)	436.8 (409.3–478.3)	0.434
Total sleep time (min)	389.0 (340.0–426.5)	386.5 (332.5–427.0)	389.8 (347.0–426.5)	0.659
% stage 1	7.2 (5.2–9.7)	7.7 (5.2–9.7)	7.0 (5.0–9.5)	0.325
% stage 2	50.1 (43.5–57.8)	48.9 (43.1–56.9)	51.1 (43.7–59.5)	0.120
% slow-wave sleep	12.4 (5.4–19.1)	12.7 (5.2–19.7)	12.4 (6.1–17.8)	0.757
% REM sleep	16.7 (11.7–21.0)	16.7 (11.7–21.8)	16.7 (11.7–20.2)	0.230
REM latency (min)	88.8 (66.0–142.0)	88.0 (62.5–136.0)	93.0 (69.0–155.5)	0.042
% wake after sleep onset	9.4 (5.4–16.1)	9.2 (5.2–15.9)	9.7 (6.2–17.1)	0.557
Number of awakenings	22 (17–30)	22 (17–29)	23 (17–31)	0.514
Micro-arousal index	9 (5–15)	9 (5–16)	9 (5–15)	0.912
Apnea–hypopnoea index	4 (1–13)	4 (1–13)	4 (2–13)	0.665
Oxygen desaturation index	3 (0–9)	3 (0–8)	3 (0–9)	0.914
Total time under 90% of SaO_2_ (min)	0.0 (0.0–8.0)	0.0 (0.0–11.0)	0.0 (0.0–6.3)	0.535
PLMs index	2 (0–9)	3 (0–8)	2 (0–13)	0.848
	**Median (P25–P75)**	**Median (P25–P75)**	**Median (P25–P75)**	**Wilcoxon test**

REM = rapid eye movement, PLMs = periodic limb movements during sleep.

## Data Availability

The data presented in this study are available on reasonable request from the corresponding author.

## Supplementary Materials

The following supporting information can be downloaded at: https://www.mdpi.com/article/10.3390/brainsci13071065/s1, Annex S1: Procedure used for outpatient recruitment of major depressed subjects; Annex S2: Description of self-questionnaires used; Annex S3: Description of sleep assessment; Annex S4: Description of the confounding factors included in the univariate analyzes. References [46,47,48,49,50,51,52,53,54,55,56,57,58,59,60,61] are cited in the supplementary materials.
