# Supplementary material for: Suicidal Ideations in Major Depressed Subjects: Role of the Temporal Dynamics of Anhedonia
Source: Brain Sci. 2023 Jul 13;13(7):1065. doi: 10.3390/brainsci13071065 (PMC10377246; doi:10.3390/brainsci13071065)
Supplement: Supplementary file 1 [file brainsci-13-01065-s001.zip › brainsci-2501349-supplementary.pdf]

Annex S1

Description of the procedure used for outpatient recruitment of major depressed subjects

All major depressed subjects recruited for this study were initially referred to the Sleep Laboratory by physicians specialized in sleep medicine after an outpatient consultation during which a preliminary assessment of their complaints related to sleep, their ongoing psychotropic/somatic treatments and their somatic/psychiatric comorbidities was systematically performed to allow a first diagnostic hypothesis. Following this initial assessment, a polysomnographic recording was programmed in all these major depressed subjects to allow an objective assessment of their sleep complaints and to exclude the presence of comorbid sleep disorders that could negatively impact mood regulation.

Annex S2

Description of self-questionnaires used

- The presence of depressive symptoms was investigated using Beck Depression Inventory (BDI-II). This scale consists of 21 items that may be scored from 1 to 3. The score may vary from 0 to 63. A score of 0-9 indicates no depression, 10-18 mild depression, 19-29 moderate depression, and 30-63 severe depression [46].
- The presence of anxious symptoms was studied using the Spielberger Anxiety Inventory. The state section assesses anxiety during the sleep laboratory whereas the trait section assesses anxiety in daily life. Each of the two sections includes 20 questions graded from 1 to 4. For each section, the score may vary from 20 to 80. The lower the final score, the lower the anxiety level and vice versa [47].
- The presence of insomnia symptoms was investigated using the Insomnia Severity Index. This index consists of 7 questions that may be scored from 0 to 4. The score may vary from 0 to 28. A score of 0-7 indicates no insomnia, 8-14 subclinical insomnia, 15-21 moderate insomnia, and 22-28 severe insomnia [48].
- Daytime sleepiness was studied using the Epworth Sleepiness Scale. This scale consists of 8 questions that may be scored from 0 to 3 and assesses daytime sleepiness in frequent situations of daily life. The score may vary from 0 to 24. A score greater than 10 indicates excessive daytime sleepiness [49].

- Temporal Pleasure Experience Scale (TEPS) is an 18-items questionnaire: 10 items measure anticipatory pleasure (anticipatory pleasure subscale [TEPS-A8T]) and 8 items measure consuming pleasure (consuming pleasure subscale [TEPS-CONS]). For each item, the subjects respond with a severity gradient ranging from 1 (completely false) to 6 (very true). The score of the anticipatory pleasure subscale varies between 10 and 60 and that of consuming pleasure between 8 and 48. The subject must describe himself as he usually is, since it is an assessment of lifelong anhedonia and not of recent change of anhedonia [21].

Annex S3

Description of the sleep assessment

In major depressed subjects recruited for this study, a specific semi-structured sleep interview based on the recommendations of the *American Academy of Sleep Medicine* was performed by a unit psychiatrist during their admission to the Sleep Laboratory in order to allow a systematic assessment of their complaints related to sleep including sleeping habits, symptoms of insomnia disorder, symptoms of sleep-related breathing disorders, symptoms of central disorders of hypersomnolence, symptoms of circadian rhythm sleep-wake disorders, symptoms of parasomnias and symptoms of sleep-related movement disorders [25]. This specific semi-structured sleep interview is a standardized procedure of the Sleep Laboratory that makes it possible to systematically research for symptoms suggestive of sleep disorders and to program polysomnographic recordings adapted to the symptoms reported by patients.

During their stay in the Sleep Laboratory, major depressed subjects included in this study benefited from a polysomnographic recording from which the data were collected for analysis. The patients went to bed between 22:00 - 24:00 and got up between 6:00 - 8:00, following their usual schedule. During bedtime hours, the subjects were recumbent and the lights were turned off. Daytime naps were not permitted.

The polysomnographic recordings performed in our unit meet the recommendations of the *American Academy of Sleep Medicine* [50]. The applied polysomnography-montage was as follows: two electro-oculogram channels, three electroencephalogram channels (Fz-Ax, Cz-Ax, and Oz-Ax, where Ax was a A1A2

mastoid reference), one submental electromyogram channel, electrocardiogram, pressure *cannula* to detect the oro-nasal airflow, finger pulse-oximetry, a microphone to record breathing sounds and snoring, plethysmographic inductive belts to measure thoracic and abdominal breathing, and anterior tibialis electrodes. Polysomnographic recordings were visually scored by specialized technicians according to the criteria of the *American Academy of Sleep Medicine* [51].

Obstructive apneas were scored if the decrease in air flow was ≥90% for at least 10 seconds whereas obstructive hypopneas were scored if the decrease in airflow was ≥30% for at least 10 seconds with a decrease in oxygen saturation of 3% or followed by microarousal [52]. The obstructive apnea-hypopnea index correspond to the total number of obstructive apneas and hypopneas divided by the period of sleep in hours. Obstructive sleep apnea syndrome was considered absent when the obstructive apnea-hypopnea index was <5/hour, mild when the obstructive apnea-hypopnea index was ≥5/hour & <15/hour and moderate to severe when the obstructive apnea-hypopnea index was ≥15/hour [53].

Periodic limb movements during sleep were scored on the basis of the following strict criteria: 1) duration between 0.5 to 10 seconds, 2) interval between 5 and 90 seconds from leg movement onset and 3) movements had to be part of a series of ≥4 consecutive movements meeting these criteria [54]. Periodic limb movement's index corresponds to the total number of periodic limb movements during sleep divided by period of sleep in hours. Moderate to severe periodic limb movements during sleep were considered to be present when the periodic limb movement's index was ≥15/hour [55]. Moreover, the diagnoses of restless legs syndrome were made according to the diagnostic criteria of the *International Restless Legs Syndrome Study Group* [56].

Finally, potential diagnoses of insomnia disorders were made according to the diagnostic criteria of the *American Academy of Sleep Medicine Work Group* whereas short sleep duration was defined as sleep time <6 hours [57,58].

Annex 4

Description of the confounding factors included in the univariate analyzes

After a review of the literature on the factors associated with suicidal ideations in major depressed subjects [16,24,59-61], the potential confounding factors included in this study were body mass index (categorized: <25 kg/m², ≥25 & <30 kg/m², ≥30 kg/m²), age (categorized: <30 years, 30-45 years, >45 years), obstructive sleep apnea syndrome (categorized: no, mild, moderate to severe), sleep duration (categorized: <6 hours, ≥6 hours), Insomnia Severity Index (categorized: <15, ≥15), anxiety symptoms (categorized: no, trait anxiety alone, state anxiety alone, trait + state anxiety), Beck Depression Inventory (BDI-II) reduced to 17 items (without items 4, 9, 12 and 21) (categorized: <15, ≥15) and as binary variables: gender, benzodiazepine receptor agonists, antidepressant therapy, other psychotropic treatments, smoking, alcohol consumption, somatic treatments, sleep movement disorders, excessive daytime sleepiness and type D personality.
